# Reliable Structures and Electronic Energies of Small Water Clusters Using Density Functional and Local Correlation Coupled Cluster Model Chemistries

**DOI:** 10.1021/acs.jpca.5c04923

**Published:** 2025-09-25

**Authors:** Benjamin T. Petty, Vance R. Fowler, Audrey Ryu, Caroline S. Glick, Carly A. Rock, Qihang Wang, Gregory S. Tschumper, George C. Shields

**Affiliations:** † Department of Chemistry, 3628 Furman University, Greenville, South Carolina 29613, United States; ‡ Department of Chemistry and Biochemistry, University of Mississippi, University, Mississippi 38677, United States; § School of Medicine, University of Mississippi Medical Center, Jackson, Mississippi 39202, United States; ∥ Department of Chemistry, 14717 Missouri University of Science and Technology, Rolla, Missouri 65409, United States; ○ Oxford High School, Oxford, Mississippi 38655, United States

## Abstract

In this paper we have assessed the ability of the domain-based local pair natural orbital (DLPNO)-CCSD­(T) method to match the explicitly correlated CCSD­(T) relative energies of (H_2_O)_*n*=3–7_ isomers along with the impact of the level of theory utilized to optimize the water cluster geometries. The benchmark structures were optimized using a 2-body:Many-body procedure in which all of the 1- and 2-body contributions are computed using CCSD­(T) while all of the higher-order many-body interactions are computed using MP2 (denoted CCSD­(T):MP2). Benchmark relative energies were computed for these CCSD­(T):MP2 optimized geometries with explicitly correlated CCSD­(T)-F12b single point energies (SPEs) using the cc-pVQZ-F12 and cc-pV5Z-F12 basis sets augmented with diffuse functions on the O atoms. The benchmark structures and energies were used to gauge the performance of less demanding computational protocols. For example, DLPNO–CCSD­(T) computations on the 31 benchmark structures with the analogous family of correlation consistent basis sets (cc-pVNZ for H and aug-cc-pVNZ for O, or simply haNZ where N = D-6) were used to estimate relative energies at the complete basis set (CBS) limit via three-point extrapolations. When compared to the CCSD­(T)-F12 benchmark data, the mean absolute differences (MADs) were ≤ 0.13 kcal/mol when triple-ζ and larger basis sets were employed. Using these DLPNO–CCSD­(T) results, we demonstrate that 2 less-demanding geometry optimization procedures, specifically the ωB97X-D density functional theory (DFT) method paired with the 6–31++G­(d,p) basis set and the density-fitted MP2 method paired with the haTZ basis set, give structures that yield nearly identical relative energies (MADs of only 0.07 and 0.02 kcal/mol, respectively, when comparing DLPNO–CCSD­(T)/ha6Z data). In addition, we show how the presence or absence of diffuse functions in the basis sets used for DLPNO–CCSD­(T) SPEs impact the quality of the relative energies. The protocol that combines ωB97X-D/6–31++G­(d,p) optimized structures with DLPNO–CCSD­(T) SPEs using triple-ζ or higher Dunning basis sets that include augmentation with diffuse functions on the oxygen atoms provides a fast and accurate method for determining the relative electronic energies of (H_2_O)_*n*=3–7_ water cluster isomers.

## Graphical abstract

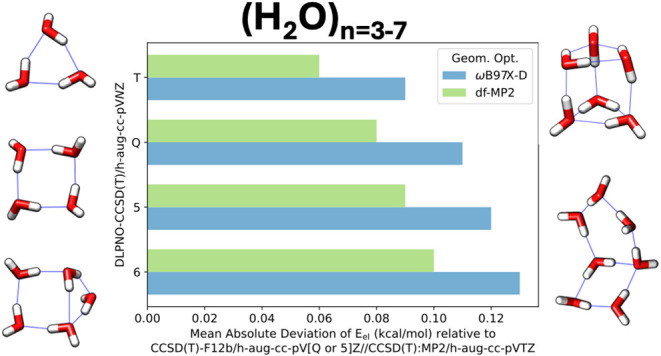

## Introduction

Water is a fascinating molecule because of its ubiquitous presence and physical properties that make life on Earth possible. The ability of water to form noncovalent, hydrogen-bonded networks is responsible for its self-interactions in the solid, liquid, and gas phases. Because intermolecular forces between gas-phase clusters of water molecules lead to fairly flat potential energy surfaces, the study of small water clusters has had a prominent role in the evolution of quantum chemistry and spectroscopic methods to understand cluster structures and energetics. The subtle differences between different isomers of a particular water cluster require highly accurate quantum chemistry techniques to capture the intermolecular forces resulting from electron correlation. The development of the coupled cluster with single, double, and perturbative triple excitations CCSD­(T) method^1–4^ has resulted in the so-called Gold Standard in quantum chemistry, where its use with large or extrapolated correlation consistent basis sets^5–7^ can converge energies to the complete basis set (CBS) limit. This is because increases in basis set size lead (slowly but eventually) to convergence of the experimental observable of interest. Typically, single point energies (SPEs) using the CCSD­(T) method are undertaken on geometries that have been generated at a lower level of theory.

Second-order Møller–Plesset perturbation theory (MP2)^8^ has been extensively used for geometries of water clusters, as it has the right balance to capture the intermolecular forces between waters.^9^ The development of correlation-consistent basis sets^5–7^ has aided in the modeling of water, and the first MP2 calculation of the C_1_ water trimer using the MP2/aug-cc-pVDZ model chemistry was published by Dunning and Xantheas in 1993.^10^ This initial study was extended in 1994 to the triple-ζ basis set for the trimer, as well as the S_4_ tetramer using the double-ζ basis set.^11,12^ Extensive studies by Xantheas and co-workers have shown that the aug-cc-pVDZ and aug-cc-pVTZ basis sets combined with MP2 theory are excellent for geometries of water clusters, and that CCSD­(T) SPEs with large or extrapolated basis sets provide converged energetic results.^13–21^ An interesting aspect of water clusters is that cooperativity effects, where homodromic arrangements of water molecules within a cluster amplify polarization, result in the most stable water structures for clusters composed of 3, 4, or 5 H_2_O fragments.^15^ In homodromic clusters, the waters are arranged such that all waters have successive donor–acceptor arrangements, and the three-body terms play a significant role in the preferential stabilities of the cyclic trimer, tetramer and pentamer isomers.^15^

Previous work by the current authors include comprehensive examinations of the water dimer and trimer potential energy surfaces using explicitly correlated computations,^22,23^ CCSD­(T)/CBS relative energies of the water hexamer isomers,^24^ reviews of reliable electronic structure methods for noncovalent clusters,^9,25^ and the development of the *N*-body:Many-body QM:QM technique for weakly bound clusters.^26–29^ This *N*-body:Many-body technique is an efficient method for reproducing CCSD­(T) energies, geometries and harmonic vibrational frequencies, and it is based on the rapid convergence of the many-body expansion for water clusters,^12,30^ such that the higher-order terms (≥*N*-body) can be included for large clusters with appropriately selected methods that have much lower computational cost.^28^ The thermodynamics for cluster formation of small water clusters has been studied by the G2,^31^ G3,^32^ CBS-QB3,^33^ and CBS-APNO^34^ model chemistries^35–37^ and structures and energies compare favorably with those using MP2/CBS as well as with experiment. As water clusters increase in size, finding the local minima becomes exceedingly more complicated, such that a brute force crawl over the entire PES is no longer possible once one exceeds five waters.^38,39^ Prediction of accurate anharmonic experimental vibrational frequencies for water clusters are useful for comparison with experiment and for calculating Gibbs free energy changes upon cluster formation.^40,41^

Experimental spectroscopy of gas-phase water clusters and quantum chemical calculations have developed together over the past four decades. Accurate predictions of water cluster isomeric structures have been essential for spectroscopists who probe cluster formation. Pioneering experiments using microwave,^42–51^ far-infrared (FIR),^51,52^ and infrared^53,54^ measurements along with theoretical studies^55–58^ allowed for a fuller understanding of the structure and tunneling present in the water dimer. The development of FIR vibration–rotation-tunneling (VRT) spectroscopy^59–61^ allowed for a detailed probe of intermolecular forces^62^ and the measurement of quantum tunneling between chiral isomers of the C_1_ trimer.^63,64^ The rotational constants obtained from this experiment were consistent with high-level quantum chemistry theory.^10,65,66^ FIR-VRT experiments helped quantify the hydrogen bonding cooperativity in the S_4_ water tetramer^11,12,67^ and the cyclic pentamer.^11,12,68^ The water hexamer is where the isomeric composition becomes quite interesting, as the PES expands from the minimum energy cyclic structures when *n* = 3–5 to the Bag, Book, Cage, and Prism three-dimensional structures.^11,12,69,70^ Site-specific infrared spectra of benzene-(H_2_O)*_n_* clusters provided evidence for noncyclic hexamer and heptamer structures^71^ as well as the D_2d_ and S_4_ cubic water octamers.^72^ A combination of experimental rotational constants extracted from VRT spectra compared with quantum Monte Carlo simulations suggested that the hexamer Cage was the most stable structure of (H_2_O)_6_.^73^ Theory suggested the Cage structure was stabilized by its relatively low zero-point energy.^74^ The S_6_ Cyclic water hexamer was observed in the unique cluster growth process in liquid helium, where the hexamer forms from the cyclic pentamer, and rapid quenching provided by liquid helium inhibits its arrangement to a potentially more stable structure such as the Cage.^17,75^ The Book isomer has also been detected by comparing the vibrational OH-stretch spectrum at cluster temperatures between 40 and 60 K with temperature dependent calculations.^76–78^ Theory has been used to extensively examine the PES for the water hexamer, and the relative energies of the S_6_ Cyclic, Book, Cage, and Prism isomers, and the lowest energy isomer is extremely sensitive to the level of electron correlation, basis set, and zero-point vibrational energy.^16,24,41,79–84^ Scaled harmonic CCSD­(T)/CBS relative Gibbs free energy benchmark calculations of eight hexamer isomers as a function of temperature predicted that one of the Book isomers, as well as the Cage and Prism, were within 0.02–0.09 kcal/mol of each other at temperatures near 0 K, with one benchmark predicting the Prism was the minimum by 0.02 kcal/mol^84^ and the other predicting the Cage by 0.09 kcal/mol.^24^ The development of a broadband Fourier transform microwave spectrometer based on chirped pulse excitation^85^ (CP-FTMW) that operates in the 2–18 GHz frequency range was an exciting development in rotational spectroscopy, as it allowed for the accurate determination of structures by isotopic substitution. Pate and co-workers identified the Cage, Prism, and Book isomers in their pulsed-jet experiment.^86^ By taking separate broadband rotational spectra in flowing argon, neon, and helium, they were able to infer that the Cage is the lowest energy isomer, and that the experimental determination of the structure was in remarkable agreement with the calculated O---O distances and other parameters from the CCSD­(T)/CBS benchmarks.^24,84,86^ While all three structures are present in the pulsed beam, the Prism structure exhibits tunnelling motion that involves the concerted breaking of two hydrogen bonds.^87^ The CP-FTMW experiment detected two isomers of the heptamer, and the experimental and calculated O---O distances were in excellent agreement.^88^

Extending this work to larger clusters is a challenge, because CCSD­(T) calculations with large basis sets require substantial computational resources and because the number of isomers for a particular cluster size increases exponentially. Thus, researchers have explored using force fields, semiempirical methods, and Density Functional Theory (DFT) to improve our ability to characterize larger cluster sizes. The problem is difficult because of the quantum nature of the cooperativity effect in hydrogen bonding of water, as already mentioned. Early work on hydrogen-bonded clusters revealed that while the semiempirical AM1 ^89^ and PM3 ^90,91^ methods were inaccurate for computing energies, the PM3 method was much better at obtaining reasonable hydrogen-bonded geometries.^92–96^ While semiempirical methods have continued to improve,^97,98^ including the development of broadly parametrized tight-binding DFT methods such as GFNn-xTB,^99–103^ DFT has become the workhorse for many computational projects. Yet DFT is not without its problems, mainly because the exact exchange functional is unknown and modeling intermolecular forces with a model based on an electron gas requires some modifications. Users of DFT typically benchmark their methods against the problem of interest and use a model chemistry that is a combination of a particular DFT functional and a basis set, as increasing the basis set size does not necessarily improve the results as it does in *ab initio* electronic structure theory. For instance, chemists studying the formation of prenucleation complexes in the atmosphere have found that the ωB97X-D/6–31++G­(d,p), M06–2*X*/6–11++G­(3df,3pd), and PW91/6–311++G­(3df,3pd) model chemistries are excellent for structure determination.^104–107^ We’ve shown that popular functionals fail to describe the stationary points on the water dimer PES,^108^ and noncyclic (H_2_O)*_n_* structures such as the tetramer pyramid and the pentamer cage are not minima on many DFT PESs.^109^ One solution that improves density functionals is to add an empirical parameter for dispersion^110^ which results in much better geometries. Significant effort has been exerted to modify the exchange-correlation functional to provide a better DFT description of hydrogen bonding, and both the Truhlar and Michaelides research groups have assessed the accuracy of DFT functionals to predict geometries and relative energies of water hexamers.^79,111^ Energetics are more difficult, as sometimes a particular DFT model chemistry locates the CCSD­(T) global minima and other times it does not, so CCSD­(T) SPEs with the aug-cc-pVTZ basis set or estimated at the CBS limit using double, triple, and quadruple-Zeta basis sets are often used to improve the energetic predictions.^104^ Martin and co-workers have done a comprehensive analysis of conventional and explicitly correlated (F12) CCSD­(T) benchmark calculations on MP2 and DFT geometry-optimized water clusters,^112^ based on our previous work using MP2/aug-cc-pVDZ geometries.^84^

Two ways to improve the ability to make faster and yet still accurate calculations of water clusters are to substitute a DFT functional for the MP2 geometry optimization step and to use a local correlation coupled cluster method with near linear scaling in place of CCSD­(T). In this work we have assessed the ability of the domain-based local pair natural orbital (DLPNO)-CCSD­(T) method in ORCA^113–126^ on ωB97X-D^127,128^ DFT geometries to model (H_2_O)*_n_* water clusters, where *n* = 3–7. The ωB97X-D/6–31++G** model chemistry is quite efficient relative to other density functionals where the model chemistry includes a triple-ζ basis set, and has proven its worth in atmospheric chemistry.^107,129^ For the DLPNO–CCSD­(T) SPEs, we have explored using the cc-pVNZ and aug-cc-pVNZ basis sets (denoted NZ and aNZ, respectively) along with a mixed prescription (heavy-aug-cc-pVNZ or simply haNZ) that only places diffuse functions on the O atoms, for N = D-6, as well as using a 4–5 inverse polynomial CBS extrapolation scheme for three successive correlation energies.^130^ Comparison of the various electronic energies on the DFT, MP2, and CCSD­(T)/many-body geometries lead to a robust comparison of these different methods.

## Methods

Identification of minimum energy clusters typically requires configurational sampling, optimizations with density functional theory, and further energy refinement with high-level wavefunction methods.^104^ In the present work, the water structures (H_2_O)_*n*=3–7_ started from previous works^83,84^ or were located after geometry optimizations of these previous structures changed the relative orientation of the dangling hydrogen atoms that do not participate in hydrogen bonding. We have denoted these conformational isomers with a lowercase “c” after each structure in the Figures. The first set of water cluster structures examined in this study were optimized in Gaussian16 Rev C01 ^131^ utilizing gradients computed with the PSI4 (release 1.2.1)^132,133^ quantum chemistry software package for density-fitted second-order Møller–Plesset perturbation theory (df-MP2)^134–136^ paired with the heavy-aug-cc-pVTZ (haTZ) correlation consistent basis set that only augments the heavier O atoms with diffuse functions (i.e., aug-cc-pVTZ for O and cc-pVTZ for H).^5,6^ A brief overview of this family of basis sets can be found in Reference 137.

A second set of optimized structures was generated in a similar fashion using analytic gradients for the *N*-body:Many-body QM:QM in-house technique^26–29^ for noncovalent clusters in which the leading terms (≤2-body) of the many-body expansion are described with the CCSD­(T) method^1–4^ whereas the remaining higher-order terms (≥3-body) are recovered with conventional MP2 computations.^8^ This 2-body:Many-body (2b:Mb) procedure is abbreviated CCSD­(T):MP2. These CCSD­(T):MP2 optimizations also employed the haTZ basis set and are expected to provide structures that are nearly identical to the CCSD­(T)/haTZ optimized geometries.^28^ The requisite CCSD­(T) gradients were computed with CFOUR version 2.1 ^138^ and Molpro version 2022.1 ^139–141^ while the MP2 gradients were obtained from Gaussian16.^131^

Explicitly correlated CCSD­(T)-F12b^142–145^ single-point energies were computed with Molpro for the CCSD­(T):MP2/haTZ optimized structures to estimate the electronic energies of the water cluster isomers near the CCSD­(T) complete basis set limit. These computations utilized the corresponding haQZ-F12 and ha5Z-F12 basis sets^146,147^ along with the default auxiliary basis sets. The CCSD­(T)-F12 energies reported in this study were obtained with ansatz F12b and without scaling the triples contributions. These are the best energies reported in this study and serve as the benchmark values for the other methods. The 1s-like core orbitals of O were frozen for all (df-)­MP2, CCSD­(T), and CCSD­(T)-F12 computations.

A third set of geometries was computed starting from the df-MP2 geometries using the ωB97X-D functional^127,128^ that includes Grimme’s dispersion correction^148^ and the 6–31++G­(d,p) basis set^149–152^ using Gaussian16 Rev B01.^131^ Tight convergence criteria was set for both the self-consistent field method and geometry optimization for all three sets of geometry optimizations, and the three sets of geometries (df-MP2, CCSD­(T):MP2, and ωB97X-D) were then used for SPEs with the domain-based local pair natural orbital coupled cluster method, DLPNO–CCSD­(T), in ORCA^113–125,153,154^ versions 4.2.1 and 5.0.3 to obtain electronic energies with the correlation consistent Dunning basis sets,^5–7^ cc-pVNZ (*N* = D,T,Q,5,6) along with the def2/J,^162^ def2/JK,^163^ cc-pVNZ/C, and aug-cc-pVNZ/C^164^ auxiliary basis sets. Specifically, we used the semicanonical triples correction (T0). The DLPNO–CCSD­(T) calculations were performed using either the default pair natural orbital (PNO) thresholds in ORCA (NormalPNO) or the more stringent TightPNO settings. These thresholds control how electron correlation is approximated and which electron pairs are retained. For NormalPNO, the energetic threshold for weak and strong electron pairs (TCutPairs) is 10^–4^; the initial domains for the PNO expansion are defined by TCutDO = 10^–2^ and TCutMKN = 10^–3^; and the minimum occupation number for PNOs to be considered significant (TCutPNO) is 3.33 × 10^–7^. For TightPNO, the thresholds are tightened to TCutPairs = 10^–5^, TCutDO = 5 × 10^–3^, TCutMKN = 10^–3^, and TCutPNO = 10^–7^. TightPNO generally provides results that are closer to canonical CCSD­(T) than NormalPNO, with the trade-off of a higher computational cost.

Three sets of calculations were completed with the ωB97X-D geometries, one using the cc-pVNZ basis sets (NZ), one with the augmented cc-pVNZ basis sets (aNZ), and one with augmentation only on the oxygen atoms (haNZ). The haNZ basis set was used for DLPNO–CCSD­(T) SPEs on the MP2 and CCSD­(T):MP2 geometries. The DLPNO correlation energies with each of these basis sets were extrapolated to the CBS limit using a 4–5 inverse polynomial CBS extrapolation scheme for three successive correlation energies,^130^ using the DTQ energies (eq 1). In eq 1, *N* is the highest angular momentum number, *E_N_* is the energy corresponding to the highest angular momentum basis set, and *a* and *b* are parameters to be determined.

1 $$E_{N}=E_{\mathrm{CBS}}+a/{\left(N+1\right)}^{4}+b/{\left(N+1\right)}^{5}$$

For the cc-pVDZ, cc-pVTZ, and cc-pVQZ basis sets, *N* = D,T,Q and solving the set of three equations for *E*_2_, *E*_3_, and *E*_4_ yields the CBS extrapolation formula (eq 2) for these three basis sets.

2 $$E_{\mathrm{CBS}}=\left(243E_{2}-2048E_{3}+3125E_{4}\right)/1320$$

To compute the total DLPNO–CCSD­(T) electronic energy, the extrapolated correlation energy was added to the HF energy of the largest basis set included in the extrapolation, as HF energies converge much faster than correlation energies to the CBS limit.^155–157^ The mean absolute differences (MAD) were computed from the minimum energy electronic energies for all methods versus the CCSD­(T)-F12b//CCSD­(T):MP2 2b:Mb energies for all 31 clusters.

## Results and Discussion

The final structures optimized at the 2b:Mb CCSD­(T):MP2 level using the haTZ basis set as well as the relative energies (Δ*E*_el_) from CCSD­(T)-F12 computations with the haQZ-F12 basis set for n = 7 and ha5Z-F12 for the smaller clusters are displayed in Figures 1–3. Many of the MP2/haTZ structures are conformational isomers (structures that differ only by rotation about a single bond) of the previously published RI-MP2 structures,^83,84^ where a dangling hydrogen has changed its position just slightly upon rotation of its water. These conformational isomers are denoted in the figures with a lower case “c” after the RI-MP2 structure name.

**Figure 1:**
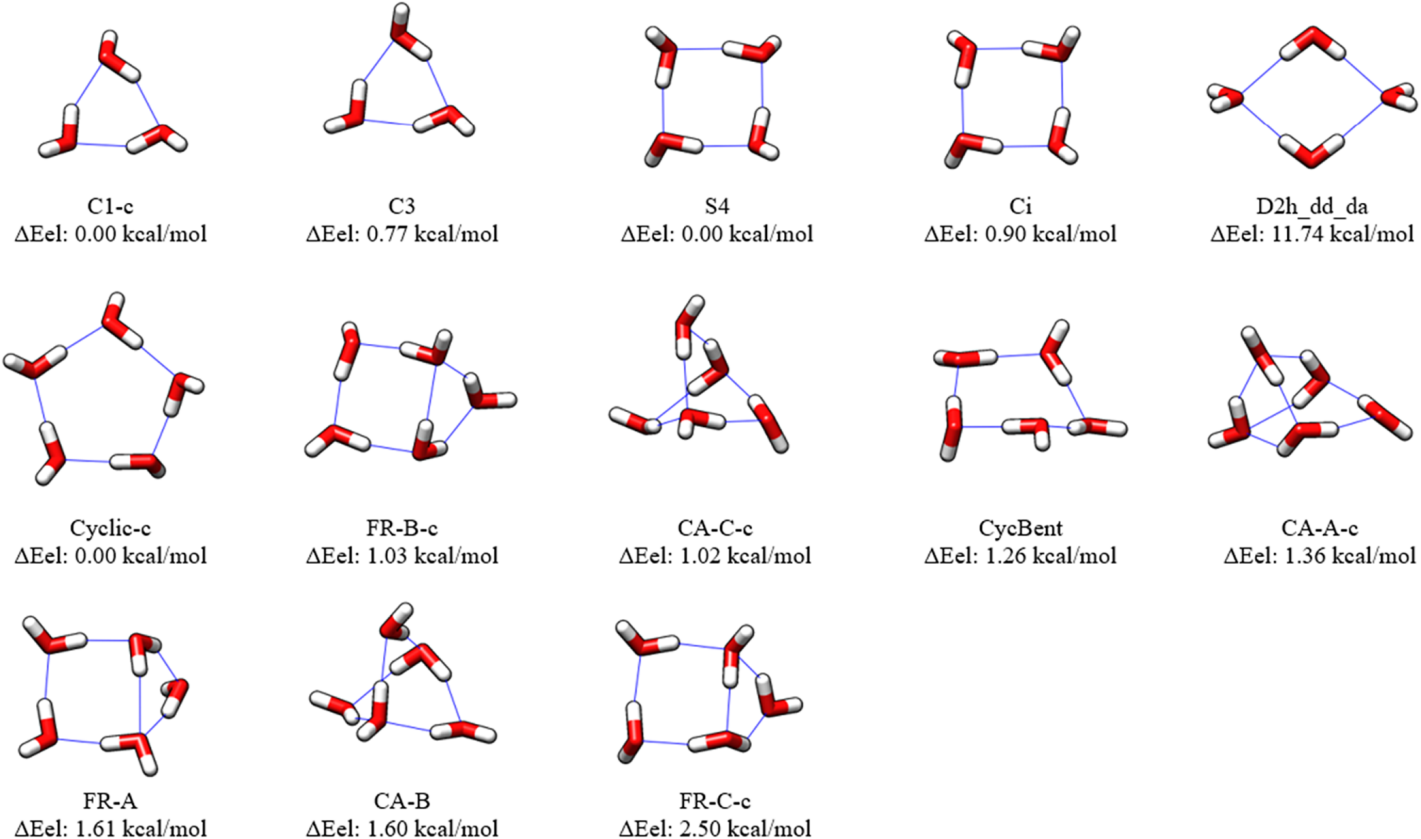
CCSD­(T):MP2/haTZ Structures and CCSD­(T)-F12b/ha5Z-F12//CCSD­(T):MP2/haTZ Relative Energies of Water Trimers, (H_2_O)_3_, Tetramers, (H_2_O)_4_, and Pentamers, (H_2_O)_5_. Conformational isomers different from the starting structures^83,84^ are denoted with a lowercase “c”.

**2:**
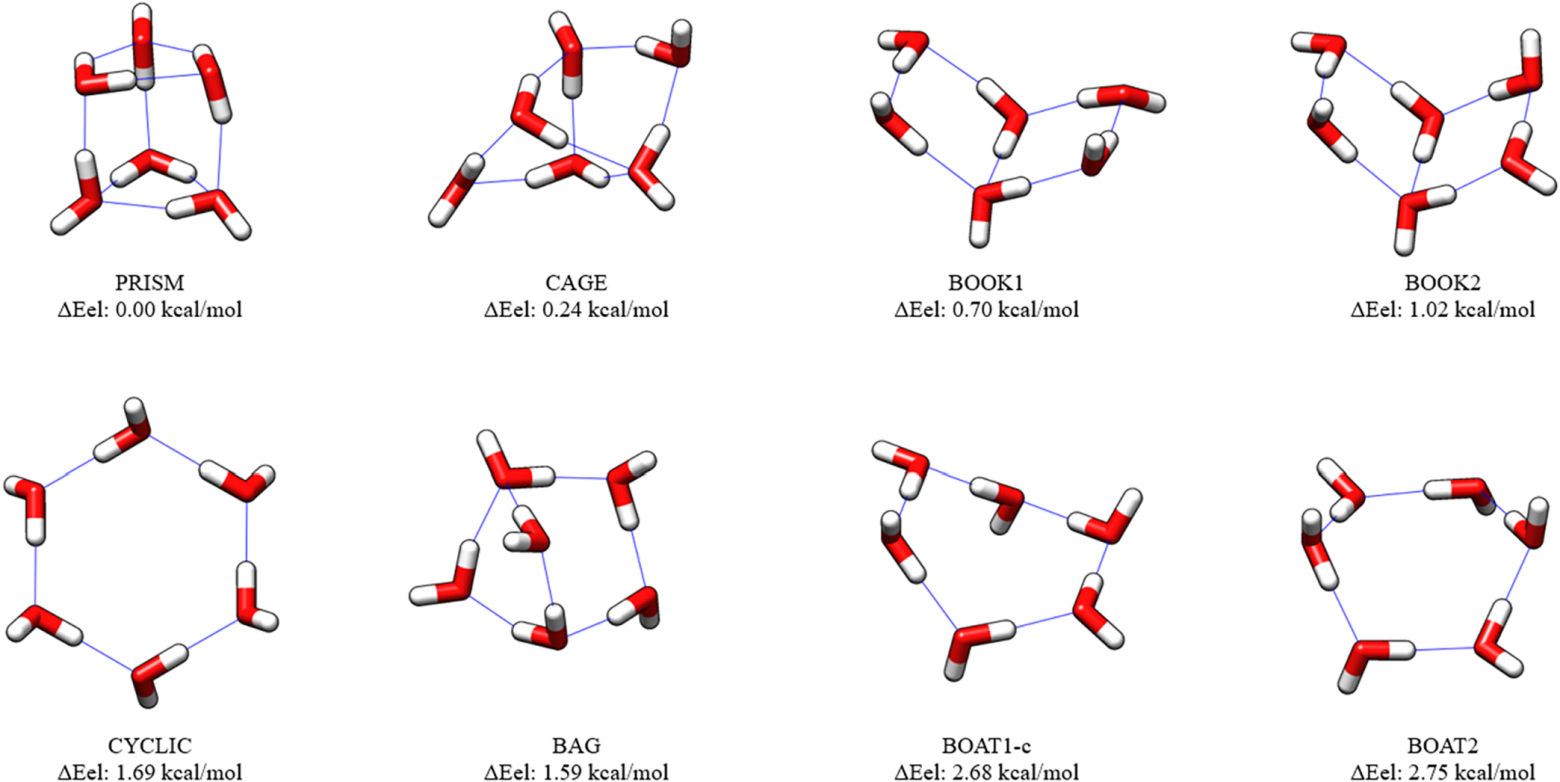
CCSD­(T):MP2/haTZ Structures and CCSD­(T)-F12b/ha5Z-F12//CCSD­(T):MP2/haTZ Relative Energies of Water Hexamers, (H_2_O)_6_. Conformational isomers different from the starting structures^83,84^ are denoted with a lowercase “c”.

**Figure 3:**
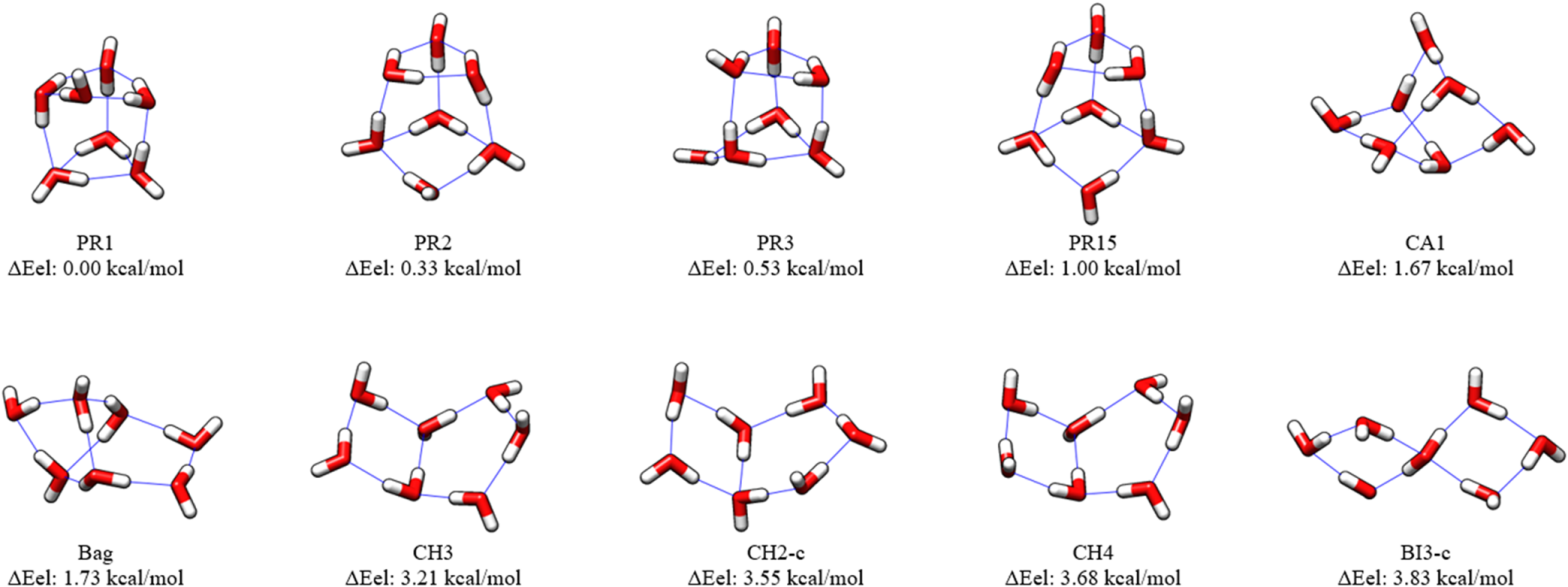
CCSD­(T):MP2/haTZ Structures and CCSD­(T)-F12b/haQZ-F12//CCSD­(T):MP2/haTZ Relative Energies of Water Heptamers, (H_2_O)_7_. Conformational isomers different from the starting structures^83,84^ are denoted with a lowercase “c”.

For each set of water clusters presented in the figures we have calculated the DLPNO–CCSD­(T) Δ*E*_el_ values using the NZ, haNZ and aNZ, *N* = D-6, series of correlation consistent basis sets for the ωB97X-D/6–31++G­(d,p), df-MP2/haTZ, and CCSD­(T):MP2/haTZ geometries. The structures and electronic energies are reported in the Supporting Information (SI).

Table 1 shows how the DLPNO–CCSD­(T) approach compares to the explicitly correlated CCSD­(T)-F12b method when the geometry is constant. In this set of calculations, all water clusters were optimized at the CCSD­(T):MP2/haTZ level. This then allows for the direct comparison of DLPNO–CCSD­(T)/haNZ// CCSD­(T):MP2/haTZ relative energies to the explicitly correlated CCSD­(T)-F12b/ha5Z-F12//CCSD­(T):MP2/haTZ values for the (H_2_O)_*n*=3–6_ water clusters and the CCSD­(T)-F12b/haQZ-F12//CCSD­(T):MP2/haTZ values for the water heptamer isomers. While tighter PNO thresholds (TightPNO) are recommended for noncovalently bound systems, the difference in TightPNO and NormalPNO energies are not as severe for hydrogen-bound systems relative to other interaction types.^119^ For water clusters (H_2_O)*_n_*, *n* = 3–7, TightPNO does not improve the agreement with our CCSD­(T)-F12b benchmark values, thus all subsequent results use the default NormalPNO thresholds in ORCA.

**Table 1:** Mean Absolute Differences (MADs in kcal/mol) of DLPNO–CCSD­(T)/haNZ and CBS Relative Electronic Energies, Using NormalPNO and TightPNO Settings in ORCA, from the Explicitly Correlated CCSD­(T)-F12b/ha5Z-F12 Values for *n* = 3–6 and haQZ-F12 for *n* = 7^a^

DLPNO–CCSD(T)/haNZ// CCSD(T):MP2/haTZ compared to CCSD(T)-F12b/ha5Z-F12// CCSD(T):MP2/haTZ	*N* = D	*N* = T	*N* = Q	*N* = 5	*N* = 6	CBS (DTQ)
NormalPNO	0.25	0.06	0.08	0.09	0.10	0.13
TightPNO	0.25	0.06	0.08	0.10	0.10	0.14

a All single point energies were computed using the CCSD­(T):MP2/haTZ optimized geometries.

For the double-ζ basis set, comparing every DLPNO–CCSD­(T)/haDZ value to the corresponding CCSD­(T)/haDZ data for all 31 (H_2_O)*_n_* clusters optimized at the CCSD­(T):MP2/haTZ level reveals that the MAD is 0.25 kcal/mol. The MAD for *N* = 2 decreases by more than a factor of 2 when the basis set size increases to *N* ≥ 3, and the MADs associated with the DTQ extrapolation is within 0.03 kcal/mol of the ha6Z value. The small range between *N* = T (0.06) and *N* = 6 (0.10) likely reflects the error bars in the DLPNO approximation relative to CCSD­(T), but it could also stem from the modest sample size of 31 clusters or a combination of both factors. These results reveal that the DLPNO method is quite reliable for comparing relative electronic energies for (H_2_O)_*n*=3–7_ clusters. We note that we have included the D_2h_ double donor-double acceptor water tetramer structure as it illustrates so well how nonhomodromic water clusters can have much higher energies, in this case 11.74 kcal/mol above the minimum according to the CCSD­(T)-F12b/ha5Z-F12 electronic energies.^15^ The corresponding NormalPNO DLPNO–CCSD­(T)/ha6Z relative energy is slightly smaller (by 0.24 kcal/mol or 2%).

Having established from Table 1 that the DLPNO–CCSD­(T) relative energies are extremely close to the explicitly correlated CCSD­(T) benchmark values, we next examine the impact of using DFT or df-MP2 to optimize the geometries of the 31 water cluster isomers examined in this work. Table 2 summarizes the energetic changes introduced by different geometry optimization protocols. DLPNO–CCSD­(T)/haNZ relative energies from SPEs on two different sets of optimized geometries (ωB97X-D/6–31++G­(d,p) and df-MP2/haTZ) were compared to those obtained with the CCSD­(T):MP2/haTZ structures. In other words, the reference values for this set of MAD data in Table 2 are the DLPNO–CCSD­(T)/haug-cc-pVNZ//CCSD­(T):MP2/haTZ relative energies. In general, the DLPNO–CCSD­(T)/haNZ relative energies computed with df-MP2/haTZ optimized structures are nearly identical to the reference values (MADs of 0.03 kcal/mol or less for N = D-6). Remarkably, the PES for the ωB97X-D/6–31++G­(d,p) geometries is also extremely close to the PES for the benchmark CCSD­(T):MP2/haTZ optimized structures, with MADs in Table 2 ≤ 0.07 kcal/mol for the N = D-6. Given the relatively low computational demands associated with ωB97X-D/6–31++G­(d,p) gradient computations, this approach may provide particularly useful prescription for the geometry optimization of larger water clusters.

**Table 2:** Mean Absolute Differences (MAD) in Electronic Energies, in kcal/mol, for DLPNO–CCSD­(T)/haNZ//ωB97X-D/6–31++G­(d,p) and DLPNO–CCSD­(T)/haNZ//df-MP2/haTZ SPEs Relative to the DLPNO–CCSD­(T)/haug-cc-pVNZ//CCSD­(T):MP2/haTZ Values for the 31 (H_2_O)_*n*=3–7_ Water Clusters Displayed in Figures 1–3

	*N* = D	*N* = T	*N* = Q	*N* = 5	*N* = 6	CBS (DTQ)
DLPNO–CCSD(T)/haNZ//ωB97X-D/6–31++G(d,p)	0.06	0.06	0.07	0.07	0.07	0.10
DLPNO–CCSD(T)/haNZ//df-MP2/haTZ	0.03	0.02	0.02	0.02	0.02	0.05

Table 3 presents the comparison of the DLPNO–CCSD­(T) SPE calculations using the cc-pVNZ, aug-cc-pVNZ, and haug-cc-pVNZ basis sets with the ωB97X-D/6–31++G­(d,p) DFT geometries relative to the DLPNO–CCSD­(T)/haug-cc-pV6Z//CCSD­(T):MP2/haTZ 2b:MB values. These results illustrate convergence as N increases as well as the effect of adding diffuse functions to the basis set. Adding diffuse functions to the oxygens (haug), or to all atoms (aug), leads to quicker convergence. MAD values converge to less than 0.1 kcal/mol for the 5Z, aQZ, and haTZ basis sets. The three extrapolations converge the MADs to less than 0.1 kcal/mol for CBS­(Q56), CBS­(aTQ5), and CBS­(haTQ5). This points to an effective strategy for determining the minimum electronic energies of larger water clusters. First, calculate the geometries with ωB97X-D/6–31++G­(d,p), then use the DLPNO–CCSD­(T) method either with cc-pVNZ (*N* = 5 or 6) or haug-cc-pVNZ (*N* = T or higher until convergence). Extrapolations at the CBS­(Q56), CBS­(aTQ5), and CBS­(haTQ5) level could also be used, and is probably good to check the convergence for larger water clusters since one would expect that larger clusters might require larger basis sets. Using these methodologies can accelerate the determination of reliable relative energies for a multitude of isomers in larger water clusters.

**Table 3:** Mean Absolute Differences (MAD) in Electronic Energies, in kcal/mol, for the DLPNO–CCSD­(T) Calculations Using Three Different Basis Sets (cc-pVNZ, aug-cc-pVNZ, and haug-cc-pVNZ) Relative to the DLPNO–CCSD­(T)/haug-cc-pV6Z//CCSD­(T):MP2/haTZ 2b:MB Values for the 31 (H_2_O)_*n*=3–7_ Water Clusters Displayed in Figures 1–3

method	*N* = D	*N* = T	*N* = Q	*N* = 5	*N* = 6	CBS (DTQ)	CBS (TQ5)	CBS (Q56)
DLPNO–CCSD(T)/NZ// ωB97X-D/6–31++G(d,p)	2.28	0.95	0.35	0.07	0.05	0.93	0.17	0.07
DLPNO–CCSD(T)/aNZ// ωB97X-D/6–31++G(d,p)	0.31	0.14	0.08	0.07	0.07	0.20	0.07	0.09
DLPNO–CCSD(T)/haNZ// ωB97X-D/6–31++G(d,p)	0.20	0.06	0.07	0.06	0.07	0.13	0.06	0.10

Importantly, this approach is not limited to pure water clusters. For instance, DLPNO–CCSD­(T)/aug-cc-pVTZ// ωB97X-D/6–31++G­(d,p)^158,159^ and CCSD­(T)/CBS//ωB97X-D/6–31++G­(d,p)^104^ have been recommended for atmospheric clusters, demonstrating the broader applicability of this methodology for efficiently obtaining reliable relative energies across a wide range of hydrogen-bound clusters.

Table 4 presents the MAD values for all of the DLPNO–CCSD­(T) calculations on the three different sets of geometries (optimized with ωB97X-D/6–31++G­(d,p), df-MP2/haTZ, CCSD­(T):MP2/haTZ) relative to the CCSD­(T)-F12b/ha5Z-F12//CCSD­(T):MP2/haTZ benchmark values for (H_2_O)_3–6_ and uses the CCSD­(T)-F12b/haQZ-F12//CCSD­(T):MP2/haTZ benchmark for the heptamer. Table S-1 contains the MAD values for all of the DLPNO–CCSD­(T) calculations on the three different geometries relative to the CCSD­(T)-F12b/haQZ-F12//CCSD­(T):MP2/haTZ benchmark values for all of the water clusters. Replacing the benchmark haQZ-F12 electronic energies for the (H_2_O)_3–6_ structures with the ha5Z-F12 electronic energies and retaining the haQZ-F12 values for the 10 heptamer structures does not change the conclusion obtained from analysis of S-1. It is apparent from the results for both sets of calculations (Table 4 and Table S-1) that to achieve convergence of around 0.1 kcal/mol with the nonaugmented basis sets on the DFT structures, that N must be extended to 5 or 6. The CBS values for the nonaugmented DTQ and TQ5 extrapolations are less accurate, a consequence of the errors stemming from the DZ and TZ SPEs. Once augmentation is employed, both the aNZ and haNZ basis sets converge much quicker. As SPE computations with the haNZ basis set are much faster than with aNZ, using it speeds up DLPNO–CCSD­(T) calculations. The MADs range from 0.10 to 0.13 kcal/mol for *N* = 6, and 0.12–0.27 kcal/mol for the augmented extrapolated methods.

**Table 4:** Mean Absolute Differences (MAD) in Electronic Energies, in kcal/mol, for DLPNO–CCSD­(T) SPEs Using Different Basis Sets for the 31 (H_2_O)_*n*=3–7_ Water Clusters Displayed in Figures 1–3.^a^

method	*N* = D	*N* = T	*N* = Q	*N* = 5	*N* = 6	CBS (DTQ)	CBS (TQ5)	CBS (Q56)
DLPNO–CCSD(T)/NZ// ωB97X-D/6–31++G(d,p)	2.18	0.86	0.26	0.08	0.13	0.84	0.24	0.15
DLPNO–CCSD(T)/aNZ// ωB97X-D/6–31++G(d,p)	0.24	0.08	0.09	0.11	0.12	0.27	0.12	0.15
DLPNO–CCSD(T)/haNZ// ωB97X-D/6–31++G(d,p)	0.28	0.09	0.11	0.12	0.13	0.17	0.13	0.16
DLPNO–CCSD(T)/haNZ// df-MP2/haTZ	0.23	0.06	0.08	0.09	0.10	0.15	0.09	0.15
DLPNO–CCSD(T)/haNZ// CCSD(T):MP2/haTZ	0.25	0.06	0.08	0.09	0.10	0.12	0.10	0.13

a Each DLPNO–CCSD­(T) energy is compared to the benchmark calculations using the explicitly correlated CCSD­(T)-F12b/ha5Z-F12//CCSD­(T):MP2/haTZ values for the (H_2_O)_*n*=3–6_ water clusters and the explicitly correlated CCSD­(T)-F12b/haQZ-F12//CCSD­(T):MP2/haTZ model chemistry for (H_2_O)_7_. Clusters are optimized with either ωB97X-D/6–31++G­(d,p), df-MP2/haTZ, or CCSD­(T):MP2/haTZ, as denoted in the Method column.

The last two rows show the MADs when the DLPNO–CCSD­(T) SPEs are carried out on the df-MP2 and CCSD­(T):MP2 geometries. The MADs are reduced for both, with values converging to 0.06–0.10 kcal/mol for the CCSD­(T):MP2 geometries with the *N* = T,Q,5,6 basis sets. The extrapolated values range from 0.09–0.15 kcal/mol for the df-MP2 geometries and 0.10–0.13 kcal/mol for the CCSD­(T):MP2 many-body geometries. Comparing the DLPNO–CCSD­(T) SPEs on the df-MP2 and CCSD­(T):MP2/haTZ geometries to the DLPNO–CCSD­(T)/haNZ values for the DFT geometries reveals that the MADs are within 0.03 kcal/mol when *N* ≥ 3. Furthermore, we note that the errors for DLPNO–CCSD­(T)/haNZ//df-MP2/haTZ are slightly smaller than the sum of MAD values from Table 1 (reflecting errors from using DLPNO and haNZ basis sets) and Table 2 (reflecting errors due to using geometries from lower-cost methods). The same trend is observed for DLPNO–CCSD­(T)/haNZ//ωB97X-D/6–31++G­(d,p). This implies that using DLPNO–CCSD­(T)/haNZ with geometries from either df-MP2/haTZ or ωB97X-D/6–31++G­(d,p) results in a favorable cancellation of error.

## Conclusions

In this work we have assessed the ability of domain-based local pair natural orbital DLPNO–CCSD­(T) single point calculations on ωB97X-D DFT geometries to model the structures and energetics of (H_2_O)*_n_* water clusters, where *n* = 3–7. We find that the ωB97X-D/6–31++G** geometries are exceptionally good relative to the df-MP2 and CCSD­(T):MP2 structures optimized with the haTZ basis set, and that the DLPNO–CCSD­(T) relative energies reproduce the explicitly correlated CCSD­(T) benchmark results quite well as long as we use appropriate basis sets.

The results reveal that the DLPNO–CCSD­(T) routine with triple-ζ or larger basis sets that are augmented with diffuse functions on oxygens is highly accurate relative to canonical CCSD­(T) calculations on geometries that are essentially equivalent to CCSD­(T)/haTZ. The Mean Absolute Differences for DLPNO–CCSD­(T)/haNZ//CCSD­(T):MP2/haTZ values relative to CCSD­(T)-F12b/ha5Z-F12//CCSD­(T):MP2/haTZ values are less than 0.1 kcal/mol when using triple-ζ basis sets (N = T) for the clusters in Figures 1–3. Furthermore, we show that changing from NormalPNO to TightPNO settings has little impact on the agreement between DLPNO–CCSD­(T) and explicitly correlated CCSD­(T) relative energies of these water clusters.

We recommend the following based on this investigation. First, the DLPNO–CCSD­(T) routine from ORCA is an excellent method for discerning the relative electronic energies of small water clusters. Second, using a basis set that has augmented functions on the oxygens and nonaugmented functions on the hydrogens (haug-cc-pVNZ) is the most efficient basis set to use for these calculations. Third, the ωB97X-D/6–31++G­(p,d) model chemistry is an excellent DFT method for calculating accurate geometries of water clusters. The use of DLPNO–CCSD­(T) SPEs on ωB97X-D/6–31++G­(p,d) geometries means that many more potential water structures can be investigated with a high degree of confidence, and the accuracy of this approach approximates benchmark CCSD­(T)-F12b/ha5Z-F12//CCSD­(T):MP2/haug-cc-pVTZ values. Finally, checking for convergence with basis sets higher than haug-cc-pVTZ for water clusters that exceed seven waters will increase confidence in the results and add to our growing understanding of how best to use electronic structure theory and DFT to investigate this fascinating system.

Our results highlight the importance of high-level electronic structure benchmarks for cluster energetics. We note that the present study focuses on electronic energies and geometry optimizations, and that the relative energetics may change at temperatures above 0 K. A free energy benchmark, where entropic and enthalpic corrections are accounted for, are important future work toward connecting theoretical predictions with experimentally observed conformers.

## Supplementary Material





## Notes

A spreadsheet that contains the Mean Absolute Differences of electronic energies relative to CCSD­(T)-F12b/haQZ-F12//CCSD­(T):MP2/haTZ, total electronic energies in hartrees for each water cluster and level of theory, relative energies in kcal/mol, and absolute and maximum errors relative to CCSD­(T)-F12b/ha­(Q or 5)­Z-F12//CCSD­(T)­MP2/haTZ.^59,72^ (XLSX)
All of the xyz files for each structure reported in this paper (ZIP)
